# (*E*)-Methyl 2-[4-(dimethyl­amino)benzyl­idene]hydrazinecarboxyl­ate at 123 K

**DOI:** 10.1107/S1600536808013950

**Published:** 2008-05-14

**Authors:** Xian-Chao Hu, Lu-Ping Lv, Wei-Wei Li, Wen-Bo Yu

**Affiliations:** a Research Center of Analysis and Measurement, Zhejiang University of Technology, Hangzhou 310014, People’s Republic of China; b Department of Chemical Engineering, Hangzhou Vocational and Technical College, Hangzhou 310018, People’s Republic of China

## Abstract

The approximately planar molecule of the title compound, C_11_H_15_N_3_O_2_, is in an *E* configuration with respect to the N=C double bond. An inter­molecular N—H⋯O hydrogen bond links the mol­ecules into a one-dimensional chain propagating in the [010] direction.

## Related literature

For general background, see: Parashar *et al.* (1988 ▶); Hadjoudis *et al.* (1987 ▶). For a related structure, see: Shi & Yuan (2006 ▶).
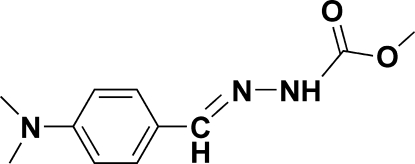


## Experimental

### 

#### Crystal data

C_11_H_15_N_3_O_2_
*M* *_r_* = 221.26
Orthorhombic, 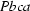

*a* = 13.051 (3) Å
*b* = 9.838 (2) Å
*c* = 18.637 (4) Å
*V* = 2392.9 (9) Å^3^
*Z* = 8
Mo *K*α radiation
μ = 0.09 mm^−1^
*T* = 123 (2) K
0.29 × 0.26 × 0.22 mm

#### Data collection

Bruker SMART CCD diffractometer
Absorption correction: multi-scan (*SADABS*; Bruker, 2002 ▶) *T*_min_ = 0.979, *T*_max_ = 0.981
19378 measured reflections
2111 independent reflections
1592 reflections with *I* > 2σ(*I*)
*R*_int_ = 0.038

#### Refinement

*R*[*F* ^2^ > 2σ(*F* ^2^)] = 0.046
*wR*(*F* ^2^) = 0.134
*S* = 1.11
2111 reflections
145 parameters
H-atom parameters constrained
Δρ_max_ = 0.23 e Å^−3^
Δρ_min_ = −0.27 e Å^−3^

### 

Data collection: *SMART* (Bruker, 2002 ▶); cell refinement: *SAINT* (Bruker, 2002 ▶); data reduction: *SAINT*; program(s) used to solve structure: *SHELXS97* (Sheldrick, 2008 ▶); program(s) used to refine structure: *SHELXL97* (Sheldrick, 2008 ▶); molecular graphics: *SHELXTL* (Sheldrick, 2008 ▶); software used to prepare material for publication: *SHELXTL*.

## Supplementary Material

Crystal structure: contains datablocks I, global. DOI: 10.1107/S1600536808013950/hb2730sup1.cif

Structure factors: contains datablocks I. DOI: 10.1107/S1600536808013950/hb2730Isup2.hkl

Additional supplementary materials:  crystallographic information; 3D view; checkCIF report

## Figures and Tables

**Table 1:** Hydrogen-bond geometry (Å, °)

*D*—H⋯*A*	*D*—H	H⋯*A*	*D*⋯*A*	*D*—H⋯*A*
N3—H3⋯O1^i^	0.86	2.16	2.976 (2)	157

Symmetry code: (i) 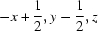.

**Fig. 1.**
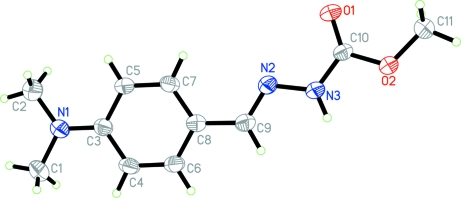
The molecular structure of (I), showing 30% probability displacement ellipsoids for the non-hydrogen atoms.

**Fig. 2.**
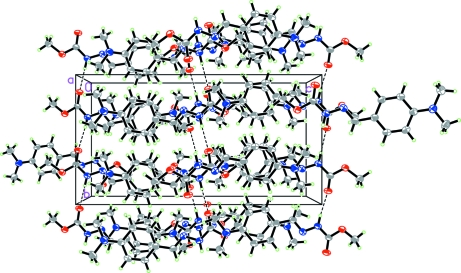
The crystal packing in (II), viewed approximately down the a axis with hydrogen bonds indicated by dashed lines.

## supplementary crystallographic information

### Comment

Benzaldehydehydrazone derivatives have received considerable attention for many years due to their pharmacological activity (Parashar *et al.*, 1988) and their photochromic properties (Hadjoudis *et al.*, 1987). As a further investigation of this type of material, the crystal structure of the title compound, C_11_H_15_N_3_O_2_, (I), is described here.

All the nonhydrogen atoms are coplanar to within ±0.1429 (14)Å (Fig. 1) in (I). The molecule is in an E conformation with respect to the N=C double bond. The bond lengths and angles of the C=N—N(H)—C groups are similar to those in related compounds (Shi *et al.*, 2006).

An intermolecular N—H···O hydrogen bond (Table 1) links the molecules into a one-dimensional chain aligned along the b direction (Fig. 2).

### Experimental

4-(Dimethylamino)benzaldehyde (14.9 g, 0.1 mol) and methyl hydrazinecarboxylate (9.0 g, 0.1 mol) were dissolved in stirred methanol (50 ml) and left for 3 h at room temperature. The resulting solid was filtered off and recrystallized from ethanol to give the title compound in 80% yield.  Colourless blocks of (I) were obtained by slow evaporation of a ethanol solution at room temperature (m.p. 452–454 K).

### Refinement

The H atoms were geometrically placed (C—H = 0.93-0.96Å, N—H = 0.86Å) and refined as riding with *U*_iso_(H) = 1.2*U*_eq_(C, N) or 1.5U_eq_(methyl C).

### Crystal data

**Table:**

C_11_H_15_N_3_O_2_	*F*_000_ = 944
*M**_r_* = 221.26	*D*_x_ = 1.228 Mg m^−^^3^
Orthorhombic, *P**b**c**a*	Mo *K*α radiation λ = 0.71073 Å
Hall symbol: -P 2ac 2ab	Cell parameters from 2111 reflections
*a* = 13.051 (3) Å	θ = 2.2–25.0º
*b* = 9.838 (2) Å	µ = 0.09 mm^−^^1^
*c* = 18.637 (4) Å	*T* = 123 (2) K
*V* = 2392.9 (9) Å^3^	Block, colourless
*Z* = 8	0.29 × 0.26 × 0.22 mm

### Data collection

**Table:**

Bruker SMART CCD diffractometer	2111 independent reflections
Radiation source: fine-focus sealed tube	1592 reflections with *I* > 2σ(*I*)
Monochromator: graphite	*R*_int_ = 0.038
*T* = 123(2) K	θ_max_ = 25.0º
ω scans	θ_min_ = 2.2º
Absorption correction: multi-scan(SADABS; Bruker, 2002)	*h* = −15→15
*T*_min_ = 0.979, *T*_max_ = 0.981	*k* = −11→11
19378 measured reflections	*l* = −21→22

### Refinement

**Table:**

Refinement on *F*^2^	Secondary atom site location: difference Fourier map
Least-squares matrix: full	Hydrogen site location: inferred from neighbouring sites
*R*[*F*^2^ > 2σ(*F*^2^)] = 0.046	H-atom parameters constrained
*wR*(*F*^2^) = 0.134	*w* = 1/[σ^2^(*F*_o_^2^) + (0.0719*P*)^2^ + 0.3078*P*] where *P* = (*F*_o_^2^ + 2*F*_c_^2^)/3
*S* = 1.11	(Δ/σ)_max_ < 0.001
2111 reflections	Δρ_max_ = 0.23 e Å^−^^3^
145 parameters	Δρ_min_ = −0.27 e Å^−^^3^
Primary atom site location: structure-invariant direct methods	Extinction correction: none

### Special details

**Table:**

Geometry. All e.s.d.'s (except the e.s.d. in the dihedral angle between two l.s. planes) are estimated using the full covariance matrix. The cell e.s.d.'s are taken into account individually in the estimation of e.s.d.'s in distances, angles and torsion angles; correlations between e.s.d.'s in cell parameters are only used when they are defined by crystal symmetry. An approximate (isotropic) treatment of cell e.s.d.'s is used for estimating e.s.d.'s involving l.s. planes.
Refinement. Refinement of *F*^2^ against ALL reflections. The weighted *R*-factor *wR* and goodness of fit *S* are based on *F*^2^, conventional *R*-factors *R* are based on *F*, with *F* set to zero for negative *F*^2^. The threshold expression of *F*^2^ > σ(*F*^2^) is used only for calculating *R*-factors(gt) *etc*. and is not relevant to the choice of reflections for refinement. *R*-factors based on *F*^2^ are statistically about twice as large as those based on *F*, and *R*- factors based on ALL data will be even larger.

### Fractional atomic coordinates and isotropic or equivalent isotropic displacement parameters (Å^2^)

**Table:**

	*x*	*y*	*z*	*U*_iso_*/*U*_eq_	
N2	0.27645 (11)	0.23338 (15)	0.56388 (7)	0.0539 (4)	
C9	0.29967 (13)	0.13610 (19)	0.60663 (9)	0.0546 (5)	
H9	0.2897	0.0469	0.5916	0.066*	
O2	0.17803 (10)	0.22366 (12)	0.38955 (6)	0.0610 (4)	
N3	0.23964 (12)	0.19224 (14)	0.49757 (7)	0.0554 (4)	
H3	0.2363	0.1072	0.4871	0.067*	
O1	0.20751 (10)	0.40732 (12)	0.45884 (7)	0.0656 (4)	
C3	0.42170 (14)	0.20049 (17)	0.81830 (9)	0.0536 (4)	
C8	0.34112 (13)	0.16094 (17)	0.67786 (9)	0.0513 (4)	
C5	0.40355 (14)	0.31063 (17)	0.77132 (10)	0.0567 (5)	
H5	0.4182	0.3986	0.7865	0.068*	
C10	0.20925 (12)	0.28641 (17)	0.44977 (9)	0.0492 (4)	
N1	0.45818 (14)	0.22037 (16)	0.88679 (9)	0.0725 (5)	
C7	0.36472 (13)	0.29041 (18)	0.70360 (10)	0.0542 (4)	
H7	0.3538	0.3652	0.6741	0.065*	
C6	0.35985 (14)	0.05251 (18)	0.72400 (10)	0.0593 (5)	
H6	0.3453	−0.0352	0.7085	0.071*	
C4	0.39921 (15)	0.07084 (18)	0.79189 (10)	0.0621 (5)	
H4	0.4111	−0.0046	0.8208	0.075*	
C11	0.14121 (16)	0.30910 (19)	0.33264 (10)	0.0667 (5)	
H11A	0.1216	0.2538	0.2925	0.100*	
H11B	0.1945	0.3707	0.3183	0.100*	
H11C	0.0830	0.3599	0.3491	0.100*	
C1	0.48056 (19)	0.1055 (2)	0.93250 (11)	0.0833 (7)	
H1A	0.5057	0.1373	0.9779	0.125*	
H1B	0.5316	0.0494	0.9101	0.125*	
H1C	0.4193	0.0534	0.9398	0.125*	
C2	0.48463 (18)	0.3522 (2)	0.91387 (11)	0.0787 (6)	
H2A	0.5090	0.3439	0.9623	0.118*	
H2B	0.4252	0.4098	0.9130	0.118*	
H2C	0.5374	0.3912	0.8845	0.118*	

### Atomic displacement parameters (Å^2^)

**Table:**

	*U*^11^	*U*^22^	*U*^33^	*U*^12^	*U*^13^	*U*^23^
N2	0.0641 (9)	0.0466 (9)	0.0509 (9)	0.0017 (6)	−0.0001 (7)	−0.0056 (7)
C9	0.0610 (10)	0.0438 (10)	0.0590 (11)	0.0027 (8)	0.0039 (8)	−0.0028 (8)
O2	0.0838 (9)	0.0425 (7)	0.0568 (8)	0.0004 (6)	−0.0117 (6)	−0.0050 (6)
N3	0.0736 (10)	0.0383 (8)	0.0544 (9)	0.0020 (6)	−0.0048 (7)	−0.0056 (6)
O1	0.0912 (9)	0.0344 (7)	0.0713 (9)	0.0016 (6)	−0.0085 (7)	−0.0070 (6)
C3	0.0545 (10)	0.0476 (10)	0.0588 (11)	0.0041 (7)	−0.0009 (8)	0.0036 (8)
C8	0.0537 (9)	0.0449 (9)	0.0555 (11)	0.0020 (7)	0.0052 (8)	0.0011 (8)
C5	0.0671 (11)	0.0407 (10)	0.0622 (11)	0.0004 (8)	−0.0026 (9)	0.0013 (8)
C10	0.0564 (9)	0.0376 (9)	0.0537 (10)	−0.0007 (7)	0.0042 (8)	−0.0053 (7)
N1	0.0990 (13)	0.0545 (10)	0.0639 (10)	0.0029 (9)	−0.0233 (9)	0.0063 (8)
C7	0.0626 (10)	0.0429 (9)	0.0571 (11)	0.0038 (8)	0.0000 (8)	0.0074 (8)
C6	0.0717 (11)	0.0401 (9)	0.0660 (12)	0.0005 (8)	−0.0003 (9)	0.0013 (8)
C4	0.0780 (12)	0.0444 (10)	0.0639 (12)	0.0062 (9)	−0.0030 (9)	0.0113 (8)
C11	0.0797 (13)	0.0577 (12)	0.0628 (12)	0.0020 (9)	−0.0115 (10)	0.0015 (9)
C1	0.0978 (16)	0.0752 (15)	0.0768 (14)	0.0021 (12)	−0.0256 (12)	0.0202 (11)
C2	0.0936 (15)	0.0716 (14)	0.0708 (13)	−0.0055 (11)	−0.0135 (11)	−0.0009 (12)

### Geometric parameters (Å, °)

**Table:**

N2—C9	1.282 (2)	N1—C2	1.434 (3)
N2—N3	1.386 (2)	N1—C1	1.445 (2)
C9—C8	1.454 (2)	C7—H7	0.9300
C9—H9	0.9300	C6—C4	1.377 (3)
O2—C10	1.344 (2)	C6—H6	0.9300
O2—C11	1.436 (2)	C4—H4	0.9300
N3—C10	1.345 (2)	C11—H11A	0.9600
N3—H3	0.8600	C11—H11B	0.9600
O1—C10	1.202 (2)	C11—H11C	0.9600
C3—N1	1.376 (2)	C1—H1A	0.9600
C3—C4	1.398 (2)	C1—H1B	0.9600
C3—C5	1.413 (2)	C1—H1C	0.9600
C8—C6	1.392 (2)	C2—H2A	0.9600
C8—C7	1.395 (2)	C2—H2B	0.9600
C5—C7	1.374 (3)	C2—H2C	0.9600
C5—H5	0.9300		
			
C9—N2—N3	114.71 (15)	C8—C7—H7	119.0
N2—C9—C8	122.00 (16)	C4—C6—C8	122.19 (17)
N2—C9—H9	119.0	C4—C6—H6	118.9
C8—C9—H9	119.0	C8—C6—H6	118.9
C10—O2—C11	116.70 (13)	C6—C4—C3	121.41 (16)
C10—N3—N2	119.44 (14)	C6—C4—H4	119.3
C10—N3—H3	120.3	C3—C4—H4	119.3
N2—N3—H3	120.3	O2—C11—H11A	109.5
N1—C3—C4	121.92 (16)	O2—C11—H11B	109.5
N1—C3—C5	121.59 (16)	H11A—C11—H11B	109.5
C4—C3—C5	116.49 (16)	O2—C11—H11C	109.5
C6—C8—C7	116.64 (16)	H11A—C11—H11C	109.5
C6—C8—C9	120.04 (16)	H11B—C11—H11C	109.5
C7—C8—C9	123.32 (16)	N1—C1—H1A	109.5
C7—C5—C3	121.32 (16)	N1—C1—H1B	109.5
C7—C5—H5	119.3	H1A—C1—H1B	109.5
C3—C5—H5	119.3	N1—C1—H1C	109.5
O1—C10—O2	124.50 (16)	H1A—C1—H1C	109.5
O1—C10—N3	126.45 (16)	H1B—C1—H1C	109.5
O2—C10—N3	109.03 (14)	N1—C2—H2A	109.5
C3—N1—C2	122.56 (16)	N1—C2—H2B	109.5
C3—N1—C1	120.36 (17)	H2A—C2—H2B	109.5
C2—N1—C1	116.83 (17)	N1—C2—H2C	109.5
C5—C7—C8	121.95 (16)	H2A—C2—H2C	109.5
C5—C7—H7	119.0	H2B—C2—H2C	109.5

### Hydrogen-bond geometry (Å, °)

**Table:**

*D*—H···*A*	*D*—H	H···*A*	*D*···*A*	*D*—H···*A*
N3—H3···O1^i^	0.86	2.16	2.976 (2)	157

Symmetry codes: (i) −*x*+1/2, *y*−1/2, *z*.
